# Anti-Inflammatory Strategy for M2 Microglial Polarization Using Retinoic Acid-Loaded Nanoparticles

**DOI:** 10.1155/2017/6742427

**Published:** 2017-08-23

**Authors:** Marta Machado-Pereira, Tiago Santos, Lino Ferreira, Liliana Bernardino, Raquel Ferreira

**Affiliations:** ^1^Health Sciences Research Centre (CICS-UBI), University of Beira Interior, Rua Marquês d'Ávila e Bolama, 6201-001 Covilhã, Portugal; ^2^Center for Neuroscience and Cell Biology (CNC), University of Coimbra, Coimbra, Portugal; ^3^Faculty of Medicine, Institute for Interdisciplinary Research of the University of Coimbra (IIIUC), Coimbra, Portugal

## Abstract

Inflammatory mechanisms triggered by microglial cells are involved in the pathophysiology of several brain disorders, hindering repair. Herein, we propose the use of retinoic acid-loaded polymeric nanoparticles (RA-NP) as a means to modulate microglia response towards an anti-inflammatory and neuroprotective phenotype (M2). RA-NP were first confirmed to be internalized by N9 microglial cells; nanoparticles did not affect cell survival at concentrations below 100 *μ*g/mL. Then, immunocytochemical studies were performed to assess the expression of pro- and anti-inflammatory mediators. Our results show that RA-NP inhibited LPS-induced release of nitric oxide and the expression of inducible nitric oxide synthase and promoted arginase-1 and interleukin-4 production. Additionally, RA-NP induced a ramified microglia morphology (indicative of M2 state), promoting tissue viability, particularly neuronal survival, and restored the expression of postsynaptic protein-95 in organotypic hippocampal slice cultures exposed to an inflammatory challenge. RA-NP also proved to be more efficient than the free equivalent RA concentration. Altogether, our data indicate that RA-NP may be envisioned as a promising therapeutic agent for brain inflammatory diseases.

## 1. Introduction

Microglia constitute the major population of resident immune-competent cells of the central nervous system (CNS), with a crucial role in brain repair, development, and homeostasis [1, 2]. After injury, these native tissue macrophages represent the first line of defense and quickly change their phenotype to secrete a large and diverse range of molecules that mediate inflammation [3, 4]. Microglia can therefore be categorized into a damaging M1 phenotype or a protective M2 phenotype [5]. M1 represents a detrimental state of microglia and is characterized by high expression of proinflammatory mediators such as nitric oxide (NO), inducible nitric oxide synthase (iNOS), interleukin- (IL-) 1*β*, IL-6, IL-12, and tumor necrosis factor- (TNF-) *α* [6]. This cell state is typically induced by interferon- (IFN-) *γ* or lipopolysaccharide (LPS), while M2 is commonly induced by IL-4 or IL-13 [7]. M2 is characterized by high levels of anti-inflammatory mediators arginase- (Arg-) 1, IL-10, transforming growth factor- (TGF-) *β*, and insulin-like growth factor- (IGF-) 1 [6]. These factors promote neuronal survival, in a process aided by the removal of neurotoxins and cellular debris and dying cells by scavenging microglia [8]. So designing an approach focused on inducing this protective phenotype in microglial cells could be advantageous in a vast array of brain disorders. In fact, inflammatory mechanisms carried out by microglia constitute a hallmark in the pathophysiology of Alzheimer's disease, Parkinson's disease, multiple sclerosis, and prion diseases [9, 10]. In order to induce a neuroprotective microglia phenotype, we propose the use of retinoic acid (RA). RA, the main biologically active derivative of vitamin A (retinol), plays an important role in neural differentiation, neuronal patterning, and axon outgrowth, which highlights its therapeutic potential for the treatment of neurodegenerative disorders [11]. Additionally, all-*trans-*RA has shown anti-inflammatory potential since retinol-deprived rats display increased reactive astrocytosis, and RA inhibits the release of inflammatory cytokines by macrophages [12–14]. Other isoforms such as 9-*cis*-RA have shown the ability to inhibit the production of NO and proinflammatory cytokines (TNF-*α* and IL-1*β*) by LPS-stimulated microglia [15, 16]. Since this molecule is unstable and rapidly degraded under physiological conditions and has low solubility in the aqueous phase, we designed a drug delivery system to ensure efficient intracellular transport and controlled release of RA, retinoic acid-loaded polymeric nanoparticles (RA-NP). We have previously demonstrated that this formulation has an approximately 2500-fold higher proneurogenic effect than the free solubilized molecule both *in vitro* and *in vivo* [17, 18]. Furthermore, we observed in a model of Parkinson's disease that RA-NP have a promising neuroprotective effect by reducing dopaminergic neuronal loss and increasing the levels of transcription factors Pitx3 and Nurr1 [19]. Recently, we also showed that RA-NP protect murine endothelial cells against an ischemic insult while promoting the release of survival and proliferative factors to neural stem cells [20]. However, the use of nanomaterials for brain delivery and/or treatment can be challenging since several elements of the nanomaterial per se can cause an adverse reaction (e.g., microglia activation) that overwhelms the positive effect that the encapsulated agent may have [21]. With this in mind, we tested the effect of RA-NP on microglial activity. Hence, we report for the first time that RA-NP have the ability to induce an M2 phenotype by inhibiting NO production and iNOS expression, by promoting Arg-1 and IL-4 expression and by modulating microglia morphology and activation, which ultimately protects neurons and restores the expression of a synaptic function marker, after an inflammatory challenge. The equivalent concentration of free RA did not induce the same effects rendering the formulation more efficiently. Collectively, our results highlight RA-NP as a relevant therapeutic agent to modulate inflammatory conditions associated to several brain inflammatory diseases.

## 2. Materials and Methods

All experiments were performed in accordance with protocols approved by national ethical requirements for animal research, the European Convention for the Protection of Vertebrate Animals Used for Experimental and Other Scientific Purposes (European Union directive number 2010/63/EU) for the care and use of laboratory animals.

### 2.1. Nanoparticle Synthesis

RA-NP were previously developed by us [17]. Briefly, free all-*trans* RA (free RA; 2% *w*/*v* in DMSO) was added to polyethylenimine (PEI; 1% *w*/*v* in borate buffer, pH 8.0). Afterwards, dextran sulfate solution (1% *w*/*v*) and 1 M zinc sulphate were added. RA-NP were then centrifuged in 5% mannitol solution at 14000*g* for 20 minutes. The resulting nanoparticles were lyophilized and stored at 4°C. Blank nanoparticles were prepared similarly but in the absence of RA. The formulation was conjugated with a green fluorophore, fluorescein isothiocyanate (FITC; 10 *μ*g/mL). The amount of RA contained in 3, 10, and 30 *μ*g/mL RA-NP is equivalent to 0.12, 0.40, and 1.20 *μ*M free RA (nonencapsulated in nanoparticles), respectively.

### 2.2. Microglia Cell Cultures and Treatment

Murine N9 microglia cell line was maintained at 37°C in a 95% atmospheric air and 5% CO_2_ humidified atmosphere in RPMI medium (Sigma-Aldrich, MO, USA) supplemented with 5 mM glucose (Sigma-Aldrich), 100 U/mL penicillin and 100 *μ*g/mL streptomycin (Life Technologies, Barcelona, Spain), and 5% fetal bovine serum (Millipore, Berlin, Germany). N9 cells were exposed to different concentrations of RA-NP and blank NP (not containing RA; 3–100 *μ*g/mL), in the presence or absence of lipopolysaccharide (LPS; 100 ng/mL) (Sigma-Aldrich) for 24 hours. Controls with free RA (0.12–10 *μ*M) (Sigma-Aldrich) and DMSO (0.01%) alone (Merck Millipore, Darmstadt, Germany) were also performed. Microglial cells were then plated at a density of 2 × 10^4^ cells per well in 24-well trays (immunocytochemical studies), plated at a density of 5 × 10^5^ cells per well in 6-well trays (NO quantification), or plated at a density of 5 × 10^3^ cells per well in 96-well trays (MTT assay).

### 2.3. 3-(4,5-Dimethylthiazol-2-yl)-2,5-diphenyltetrazolium Bromide (MTT) Assay

After cell treatments, MTT solution (5 ng/mL) was added to cells for 4 hours. Afterwards, 10% sodium dodecyl sulfate (SDS) in 0.01 M phosphate-buffered saline (PBS) was added to dissolve formazan crystals. Cytotoxicity was determined by measuring optical density at 630 nm. Data were normalized relative to control (untreated cells).

### 2.4. Griess Assay

After cell treatments, lysis mixture solution (137 mM NaCl, 20 mM Tris–HCl, 1% Triton X-100, 10% glycerol, 1 mM phenylmethylsulfonyl fluoride, 10 *μ*g/mL aprotinin, 1 *μ*g/mL leupeptin, and 0.5 mM sodium vanadate, all from Sigma-Aldrich; pH 8.0) was added to cells. Griess reagents were added (1 : 1) to each well: 0.1% N-1-naphthylenediamine dihydrochloride and 1% sulfanilamide in 5% phosphoric acid (Promega, WI, USA). NO production was determined through the formation and accumulation of its stable metabolite product nitrite (NO_2_) by measuring optical density at 540 nm in an enzyme-linked immunosorbent assay plate reader (SPECTRA max 384 Plus, Molecular Devices). The total amount of protein was quantified using the bicinchoninic acid (BCA) assay (Thermo Scientific, MA, USA). Data were normalized to control.

### 2.5. Immunocytochemistry

Cells were fixed with 4% paraformaldehyde (PFA) (Sigma-Aldrich), washed with PBS, and placed in blocking solution (0.3% BSA) in 0.1% Triton X-100 solution (Sigma-Aldrich) for 20 minutes at room temperature (RT) to prevent nonspecific binding. Cells were incubated overnight at 4°C in a primary antibody solution and then in the corresponding secondary antibody solution for 1 hour at RT. The following antibodies were used: purified mouse anti-iNOS/NOS type II (1 : 500) (BD Transduction Laboratories, BD Biosciences), purified rat anti-mouse IL-4 (1 : 100) (BD Biosciences), and purified mouse anti-arginase I (1 : 400) (BD Transduction), Alexa Fluor 546 donkey anti-mouse, Alexa Fluor 488 donkey anti-mouse, and Alexa Fluor 594 donkey anti-mouse (1 : 200) (all from Life Technologies). Nuclei were stained with Hoechst 33342 (4 *μ*g/mL) (Molecular Probes, OR, USA). Cell preparations were mounted in Dakocytomation fluorescent medium (Dakocytomation Inc., CA, USA), and corresponding images were acquired by confocal microscopy (LSM 710, Carl Zeiss, Gottingen, Germany). Images were uploaded to the ImageJ software (NIH, Bethesda, MD), the background was subtracted, and the mean intensity of each fluorescent marker, that is, the average gray value within a selected field was measured. The mean gray value is calculated by the sum of the gray values of all the pixels divided by the total number of pixels. Data was normalized by the number of cells (nuclei) per field.

### 2.6. Organotypic Hippocampal Slice Cultures

Slice cultures were obtained from 7-day-old C57BL/6J mice as previously described by us [22]. Briefly, brains were removed to isolate both hippocampi in Gey's Balanced Salt Solution (GBSS) (Biological Industries, Kibbutz Beit-Haemek, Israel) under sterile conditions. Hippocampi were cut into 350 *μ*m-thick slices using a McIlwain tissue chopper and transferred to 0.4 *μ*m porous insert membranes (Millipore Corp., Bedford, MA), which were placed in six-well plates containing culture medium (composed of 25% heat-inactivated horse serum, 50% Opti-MEM minimal essential medium, 25% Hank's Balanced Salt Solution (HBSS), 25 nM D-glucose (Merck Millipore, Darmstadt, Germany), and 50 U/mL penicillin and 50 *μ*g/mL streptomycin) (all from Invitrogen, CA, USA). Each membrane contained six slices and was kept in a humidified atmosphere (5% CO_2_) at 37°C, and media were refreshed every 2 days. After 2 weeks, slice cultures were exposed to LPS and cotreated with RA-NP or free RA for 24 hours (immunohistochemistry studies) or for 3 days (western blot analysis). To assess organotypic slice culture viability, slices were treated with 3 *μ*M propidium iodide (PI, Sigma-Aldrich) for 24 hours prior to fixation. Photomicrographs were recorded using a digital camera (Axiocam HRC, Carl Zeiss, Gottingen, Germany) coupled to an Axioskop 2 Plus fluorescent microscope (Carl Zeiss). The percentage of PI-positive cells in organotypic slices was calculated from the mean fluorescence intensity values as described in the previous section.

### 2.7. Immunohistochemistry and 3D Morphometric Analysis of Microglia

Organotypic cultures were fixed with PFA for 6 hours at 4°C and permeabilized with 0.5% Triton X-100 in PBS overnight at 4°C. After being washed, slices were placed in blocking solution (2% heat-inactivated horse serum and 0.3% Triton X-100 in PBS) for 1 hour at RT. Slice cultures were incubated overnight at 4°C in a primary rat monoclonal anti-CD11b (1 : 500) (AbD Serotec, Oxford, UK) antibody solution containing 0.3% Triton X-100 and in the corresponding secondary antibody Alexa Fluor 488 donkey anti-rat (1 : 500) (Life Technologies) for 2 hours at RT. Nuclei were stained with Hoechst 33342 (4 *μ*g/mL) (Molecular Probes). Cell preparations were mounted in Dakocytomation fluorescent medium (Dakocytomation Inc.), and corresponding Z-stacks were acquired by confocal microscopy (LSM 710). Z-stacks were uploaded to the FIJI-ImageJ software (NIH, Bethesda, MD), microglial morphology was quantified, and the Simple Neurite Tracer (SNT) plugin was used to acquire morphometric data (number and length of microglial processes and their analysis) as described by others [23, 24].

### 2.8. Western Blot Analysis

Slices were lysed using RIPA lysis buffer (0.15 M NaCl, 0.05 M Tris, 5 mM ethylene glycol tetraacetic acid, 1% Triton X-100, 0.5% deoxycholic acid, 0.1% sodium dodecyl sulphate, and 10 mM dichlorodiphenyltrichloroethane), containing a cocktail of proteinase inhibitors (Roche Diagnostics Ltd., Mannheim, Germany). Total protein concentration was determined using the BCA assay (Thermo Scientific). All samples were loaded onto 8 or 12% bisacrylamide gels (Applichem, Darmstadt, Germany). Proteins were separated by sodium dodecyl sulphate-polyacrylamide gel electrophoresis (Mini-PROTEAN® Tetra Handcast, Bio-Rad, CA, USA), in a Tris-glycine running solution (pH 8.3; Acros Organics, Geel, Belgium) at RT and were transferred to a polyvinylidene difluoride membrane (GE Healthcare, Little Chalfont, UK) using Towbin transfer buffer (25 mM Tris, 192 mM glycine, and 20% methanol; pH 8.3) through a semidry transfer (Trans-Blot® Turbo™ Blotting System, Bio-Rad). All membranes were blocked using Tris-buffered saline containing 0.1% Tween 20 (TBS-T; Sigma-Aldrich) and 0.1% porcine gelatin (Sigma-Aldrich) and then incubated overnight at 4°C with primary antibodies. Primary antibodies used were mouse anti-PSD-95 (1 : 1000; Millipore), rabbit anti-enolase (1 : 500; St John's Laboratories, London, UK), and mouse anti-tubulin (1 : 5000; Sigma-Aldrich) as endogenous control, all diluted in TBS-T. Membranes were incubated with goat anti-mouse secondary antibody (1 : 5000; Santa Cruz Biotechnology, Inc., CA, USA) and anti-rabbit antibody (1 : 1000; Thermo Scientific), all diluted in TBS-T at RT for 2 hours. Finally, protein levels were detected by enhanced chemiluminescence (ECL) exposure (ChemidocTMMP imaging system (BioRad Laboratories, CA, USA)) and densitometric analyses using the ImageJ software (NIH).

### 2.9. Statistical Analysis

Experimental conditions were performed in duplicate from at least three sets of independent experiments (*n*), unless stated otherwise. For immunocytochemistry analysis, 5 microscopy fields were acquired per coverslip (with approximately 40 cells per field). Statistical significance was determined using Student's *t*-test or one-way analysis of variance followed by Dunnett's or Bonferroni's multiple comparison test and was considered relevant for *p* values < 0.05. Data are demonstrated as a mean ± standard error of mean (SEM).

## 3. Results

### 3.1. RA-NP Do Not Compromise Microglial Cell Viability

**Figure 1:**
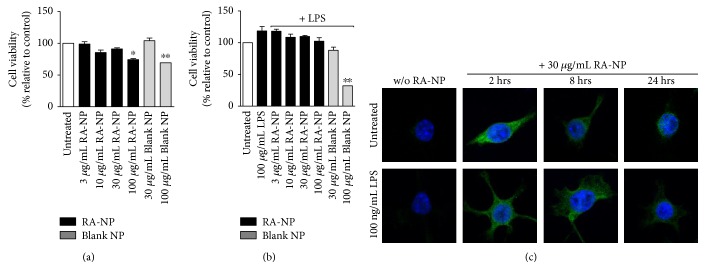
RA-NP did not compromise microglia cell viability. (a) Microglial cells were treated with RA-NP and blank NP (24 hours) to assess toxicity, using MTT assay. Cytotoxicity was induced at concentrations starting at 100 *μ*g/mL (*n* = 3; ^∗^*p* < 0.05 and ^∗∗^*p* < 0.01 compared to untreated cells). (b) LPS (100 ng/mL) did not potentiate cytotoxicity; only blank nanoparticles (100 *μ*g/mL) in the presence of LPS significantly compromised viability (*n* = 3; ^∗∗^*p* < 0.01 compared to untreated cells). (c) RA-NP (30 *μ*g/mL) internalization by microglial cells was observed by confocal microscopy over the course of 24 hours, in the absence (top row) or presence of LPS (bottom row). Scale bar 10 *μ*m.

The impact of RA-NP on microglial viability was evaluated by MTT reduction assay (Figure 1(a)). Treatments up to 30 *μ*g/mL RA-NP and blank NP did not disrupt microglia cell metabolic activity (3RA-NP = 98.90 ± 3.61%, 10RA-NP = 85.41 ± 3.95%, 30RA-NP = 91.14 ± 1.78%, and 30blank NP = 104.10 ± 4.22%; *n* = 3), compared to control. In the presence of LPS (100 ng/mL), only 100 *μ*g/mL blank nanoparticles (100blankNP = 32.10 ± 1.95%) significantly compromised microglial viability (LPS = 118.40 ± 7.00%, 3RA-NP = 118.00 ± 3.23%, 10RA-NP = 108.60 ± 4.96%, 30RA-NP = 109.80 ± 1.80%, 100RA-NP = 102.30 ± 3.23%, and 30blank NP = 88.03 ± 5.06%; *n* = 3) (Figure 1(b)). Thus, to assess nanoparticle internalization over the course of time (for 24 hours), the highest nontoxic concentration of RA-NP was chosen (30 *μ*g/mL). The incorporation of FITC (green fluorescent molecule) in the formulation allowed visualization by confocal microscopy. RA-NP internalization was performed in the presence or absence of the proinflammatory stimulus, and the maximal signal was obtained at 2 hours after treatment, in either case (Figure 1(c)).

### 3.2. RA-NP Prevent NO Production and Decrease iNOS Expression by Microglial Cells after an Inflammatory Challenge

**Figure 2:**
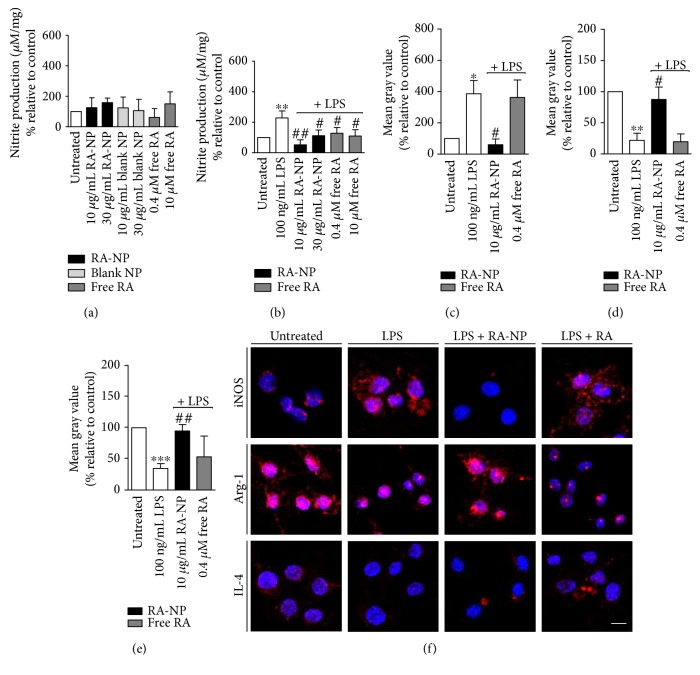
RA-NP induced an M2 microglial phenotype under inflammatory challenge. (a) None of the cell treatments (RA-NP, blank NP or free RA) affected NO levels in the absence of a stimulus. (b) RA-NP (10 *μ*g/mL) and free RA (0.4 and 10 *μ*M) inhibited NO production in the presence of 100 ng/mL LPS (24 hours) (*n* = 4; ^∗∗^*p* < 0.01 compared to untreated cells; ^#^*p* < 0.05, ^##^*p* < 0.01 compared to LPS). (c) RA-NP (10 *μ*g/mL) decreased LPS-induced iNOS expression while free RA had no effect (*n* = 3–6; ^∗^*p* < 0.05 compared to untreated cells, ^#^*p* < 0.05 compared to LPS). (d) RA-NP (10 *μ*g/mL) increased LPS-inhibited Arg-1 expression. Free RA (0.4 *μ*M) had no effect (*n* = 3–6; ^∗∗^*p* < 0.01 compared to untreated cells, ^#^*p* < 0.05 compared to LPS). (e) RA-NP (10 *μ*g/mL) increased IL-4 expression while free RA had no effect (*n* = 3–6; ^∗∗∗^*p* < 0.001 compared to untreated cells, ^##^*p* < 0.01 compared to LPS). (f) Representative confocal images depicting expression of iNOS, Arg-1, and IL-4 after cell treatments (in red; top, middle, and bottom panels, resp.). Nuclear staining in blue. Scale bar 10 *μ*m.

Activated M1 microglia release a large range of proinflammatory and neurotoxic mediators, including cytokines (e.g., TNF-*α* and IL-1*β*), free radicals (e.g., NO and superoxide), and other metabolites [25]. Accordingly, we quantified the levels of NO produced by microglial cells in the absence or presence of the proinflammatory stimulus (100 ng/mL LPS, 24 hours) (Figures 2(a) and 2(b), resp.). Using the Griess assay, we demonstrated that none of the treatments, including blank NP and free RA per se, changed basal NO production (Figure 2(a)). LPS-stimulated cells produced approximately two times more NO as compared to control (LPS = 228.20 ± 22.97%; ^∗∗^*p* < 0.01, *n* = 4). After RA-NP treatment, this effect was prevented since the formulation (10 *μ*g/mL) could significantly inhibit NO production and more evidently than 0.4 *μ*M free RA (LPS + 10RA-NP = 125.20 ± 37.61% and LPS + 0.4RA = 128.30 ± 16.54%; ^##^*p* < 0.01, ^#^*p* < 0.05, *n* = 3–5). Other concentrations of RA-NP and free RA were less efficient. Thus, subsequent experiments were performed with the lowest concentration of RA-NP and free RA (10 *μ*g/mL RA-NP and 0.4 *μ*M free RA). Then, we tested whether RA-NP affected the synthesis of inducible nitric oxide synthase (iNOS), the main enzyme expressed by microglia that is responsible for NO production. By immunocytochemical studies, we observed that cells exposed to an inflammatory environment had the strongest expression of iNOS (LPS = 386.20 ± 85.08%; ^∗^*p* < 0.05, *n* = 3). When these cells were treated with RA-NP, iNOS expression was inhibited (LPS + 10RA-NP = 60.53 ± 36.03%; ^#^*p* < 0.05, *n* = 3) compared to LPS-stimulated cells. Free RA did not change significantly iNOS expression in the presence of LPS (LPS + 0.4RA = 362.90 ± 111.70%; *n* = 3) (Figures 2(c) and 2(f), top panel). These results support the increased effectiveness of our formulation since 10 *μ*g/mL RA-NP contain the equivalent to 0.4 *μ*M of free RA.

### 3.3. RA-NP Increase Arg-1 and IL-4 Expression by Microglial Cells in an Inflammatory Environment

To further elucidate the effect of RA-NP under inflammatory conditions, we evaluated the expression of classic anti-inflammatory mediators of the M2 phenotype (namely, Arg-1 and IL-4) [6, 26]. By immunocytochemical studies, we observed that cells treated with LPS demonstrated a weak expression of Arg-1 (LPS = 22.01 ± 11.34%; ^∗∗^*p* < 0.01, *n* = 4), and when cells were cotreated with 10 *μ*g/mL RA-NP, Arg-1 expression was almost completely restored (LPS + 10RA-NP = 87.54 ± 19.81%; ^#^*p* < 0.05, *n* = 3). A low Arg-1 expression was obtained in free RA-treated cells under inflammatory conditions, similarly to LPS-treated cells (LPS + 0.4RA = 19.56 ± 12.52%; *n* = 3) (Figures 2(d) and 2(f), middle panel). Similar results were obtained for IL-4 expression (LPS = 34.60 ± 7.80%, LPS+ 10RA-NP = 94.70 ± 10.30%, and LPS + 0.4RA = 53.27 ± 33.34%; ^∗∗∗^*p* < 0.001, ^##^*p* < 0.01, *n* = 3–5) (Figures 2(e) and 2(f), bottom panel). These results suggest again the efficacy of RA-NP compared to the equivalent free RA concentration and its ability to promote a protective M2 phenotype on microglial cells.

### 3.4. RA-NP Modulate Microglia Activation and Morphology in LPS-Treated Hippocampal Slice Cultures

**Figure 3:**
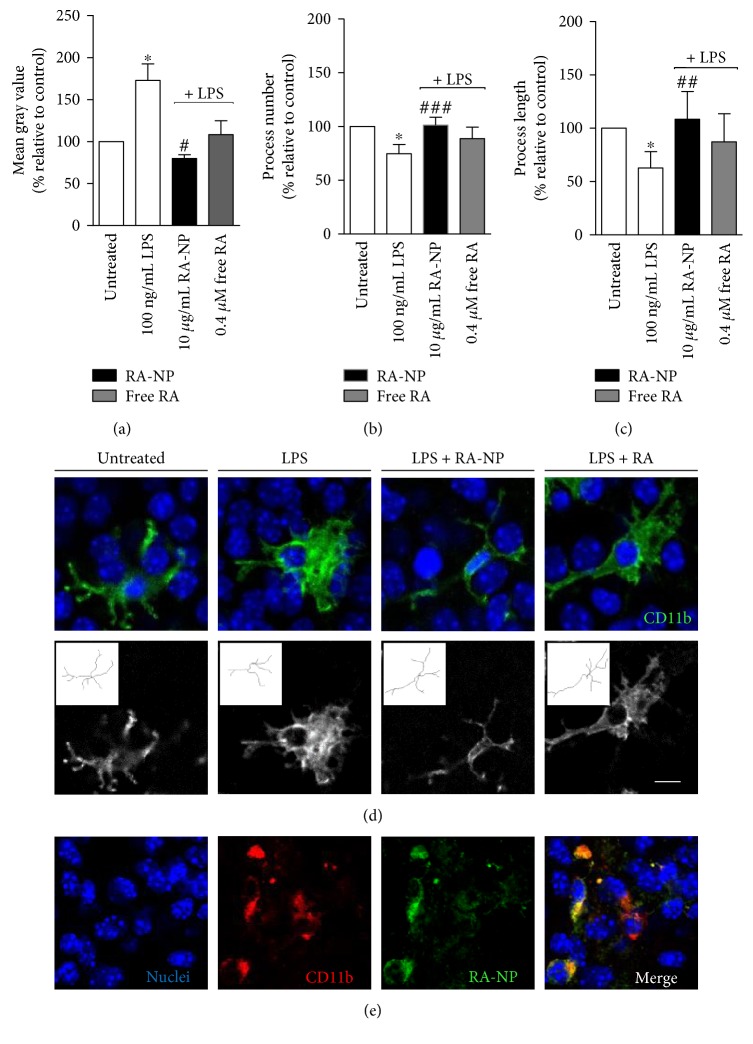
RA-NP modulated microglia activation and morphology in LPS-treated hippocampal slice cultures. Murine organotypic hippocampal slice cultures (P7) were treated with RA-NP (10 *μ*g/mL) or free RA (0.4 *μ*M), and their effect on cell morphology was quantified in an inflammatory context (100 ng/mL LPS, 24 hours). (a) RA-NP treatment significantly reduced cell bodies; free RA (0.40 *μ*M) had no effect (*n* = 4; ^∗^*p* < 0.05 compared to untreated cells; ^#^*p* < 0.05 compared to LPS). (b) RA-NP treatment (10 *μ*g/mL) significantly promoted a higher number of microglial processes while free RA (0.40 *μ*M) had no effect (*n* = 4; ^∗^*p* < 0.05 compared to untreated cells, ^###^*p* < 0.001 compared to LPS). (c) RA-NP treatment (10 *μ*g/mL) significantly promoted an increase in length of microglial processes. Free RA had no effect (*n* = 4; ^∗^*p* < 0.05 compared to untreated cells, ^##^*p* < 0.01 compared to LPS). (d) Representative brain slices were stained for CD11b (green; top panel), and skeletonized microglial cells are shown in the bottom panel. Nuclear staining in blue. (e) Microglial cells (in red) internalized RA-NP (in green). Colocalization is highlighted in the merged image. Nuclear staining in blue. Scale bar 10 *μ*m.

To further characterize the ability of RA-NP to modulate microglial activity, we used an ex vivo organotypic hippocampal slice culture model. We performed CD11b (a surface microglial marker) immunostaining to analyze microglia morphology since it is an important hallmark of its polarization [27]. As expected, 10 *μ*g/mL RA-NP changed microglia morphology from enlarged and amoeboid (LPS-activated state) to a small and ramified one, in an inflammatory context, while free RA had no effect (LPS = 172.80 ± 19.81%, LPS + 10RA-NP = 79.86 ± 4.51%, and LPS + 0.4RA = 108.20 ± 16.54%; ^∗^*p* < 0.05, ^#^*p* < 0.05; *n* = 4) (Figure 3(a)). RA-NP (10 *μ*g/mL) also promoted an increase in the number of microglial processes (LPS = 74.79 ± 4.22%, LPS + 10RA-NP = 101.30 ± 3.65%, and LPS + 0.4RA = 88.76 ± 5.33%; ^∗^*p* < 0.05, ^###^*p* < 0.001; *n* = 4) (Figure 3(b)), as well as in their length (LPS = 62.77 ± 7.66%, LPS + 10RA-NP = 108.40 ± 13.04%, and LPS + 0.4RA = 87.24 ± 13.19%; ^∗^*p* < 0.05, ^##^*p* < 0.01; *n* = 4) (Figure 3(c)), compared to LPS-activated state. Representative images of these effects are depicted in Figure 3(d). RA-NP or free RA alone had no significant effect (data not shown). We also evaluated which population of brain parenchymal cells could display colocalization with RA-NP (in green) and found distinct internalization of the formulation by microglial cells (in red). Other phenotypes, namely, neurons and astrocytes did not show internalization after 24 hours (Figure 3(e)), which does not exclude RA-NP uptake by these cell types at other time points or concentrations.

### 3.5. RA-NP Promote Hippocampal Slice Culture Viability, Particularly Neuronal Survival, after an Inflammatory Challenge

**Figure 4:**
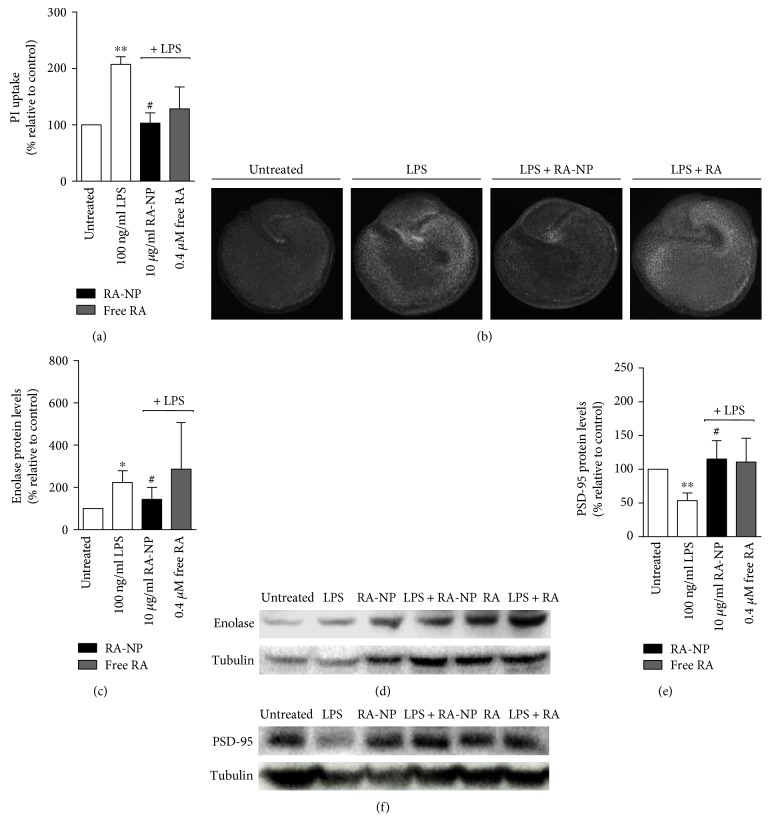
RA-NP promoted tissue viability and enhanced neuronal protection after an inflammatory challenge. (a) RA-NP (10 *μ*g/mL) protected from LPS-induced toxicity while free RA (0.40 *μ*M) had no effect (*n* = 3; ^∗∗^*p* < 0.01 compared to untreated cells, ^#^*p* < 0.05 compared to LPS). (b) Representative images depicting cell death on organotypic hippocampal slice cultures. Slices were counterstained with propidium iodide (PI). (c) RA-NP (10 *μ*g/mL) significantly counteracted the LPS effect by decreasing enolase levels. (*n* = 3; ^∗^*p* < 0.05 compared to untreated cells, ^#^*p* < 0.05 compared to LPS). (d) RA-NP (10 *μ*g/mL) significantly inhibited the LPS effect by increasing PSD-95 levels (*n* = 3; ^∗∗^*p* < 0.01 compared to untreated cells, ^#^*p* < 0.05 compared to LPS). Free RA (0.40 *μ*M) had no effect on both markers. Representative images depicting the effect of RA-NP treatment on neuronal damage (e) and synaptic function (f). Additional controls with RA-NP alone and free RA are also shown.

The inhibition of microglial M1 activation constitutes a valid therapeutic strategy to revert neurodegenerative and inflammatory disorders [3]. Thus, we assessed the role of RA-NP on neural cell viability, possibly linked to the anti-inflammatory effects observed previously. Slice cultures were treated with RA-NP or free RA, in the presence or absence of LPS. We observed that 10 *μ*g/mL RA-NP significantly reduced LPS-induced cell death while free RA had no effect (LPS = 207.40 ± 13.58%, LPS + 10RA-NP = 103.10 ± 18.35%, and LPS + 0.4RA = 128.50 ± 38.92%; ^∗∗^*p* < 0.01, ^#^*p* < 0.05, *n* = 3), compared to control (Figure 4(a)). Representative images depicting cell death on organotypic hippocampal slice cultures are shown in Figure 4(b). RA-NP or free RA alone had no significant effect (data not shown). We also assessed the role of RA-NP on neuronal injury by measuring enolase expression, a marker of neuronal damage [28]. We observed that 10 *μ*g/mL RA-NP significantly reduced LPS-induced neuronal cell injury while free RA had no effect (LPS = 223.60 ± 31.81%, LPS + 10RA-NP = 143.80 ± 32.25%, and LPS + 0.4RA = 286.10 ± 127.90%; ^∗^*p* < 0.05, ^#^*p* < 0.05, *n* = 3), compared to control (Figure 4(c)). Representative images depicting the effect of RA-NP treatment on neuronal damage and additional controls with RA-NP alone and free RA are also shown in Figure 4(e).

### 3.6. RA-NP Modulate Hippocampal Synaptic Function after an Inflammatory Challenge

Finally, M1 microglial cells secrete cytotoxic factors that are detrimental to neurons and compromise synaptic function [29, 30]. We assessed the expression of PSD-95, a postsynaptic protein, to evaluate if the formulation could reverse LPS-induced neuronal dysfunction. In fact, our results demonstrated that LPS decreased PSD-95 levels (LPS = 53.57 ± 6.48; ^∗∗^*p* < 0.01, *n* = 3) and, under inflammatory conditions, RA-NP significantly counteracted this effect (LPS + 10RA-NP = 115.10 ± 15.67; ^#^*p* < 0.05, *n* = 3) (Figure 4(d)). Free RA had no significant effect on this synaptic target. Representative images depicting the effect of RA-NP treatment on synaptic function and additional controls with RA-NP alone and free RA are also shown in Figure 4(f).

## 4. Discussion

Microglia morphology and activity are directly implicated in the efficacy of the repair process. When activated, microglia cells respond as phagocytes and are able to release inflammatory mediators (e.g., cytokines, chemokines, and reactive oxygen species) that, in excess, may disrupt the blood-brain barrier and influence neurogenesis and neuronal survival [25, 31, 32]. In this sense, the development of effective strategies that are able to modulate cell responses under these adverse conditions is an emerging need. For that reason, studies have investigated the application of anti-inflammatory molecules (e.g., glucocorticoids, minocycline, vitamins, growth factors, and endocannabinoids) as a means to repress M1 microglial activation and favor a neuroprotective effect [3]. A limitation of these compounds is their usual low systemic bioavailability and rapid degradation or vulnerability to light, pH, and temperature changes. Hence, nanomaterials convey appropriate delivery vehicles that enhance the therapeutic potential of their load [33].

Thus, we propose the use of RA-NP as a means to modulate microglia response towards an anti-inflammatory and neuroprotective phenotype (M2).

In this work, we demonstrated for the first time that RA-NP can act as a key modulator of the inflammatory reaction, acting dually as an M1 microglial activation repressor and M2 inducer. Firstly, we confirmed that RA-NP did not compromise microglia cell viability in a wide range of biocompatible concentrations (up to 30 *μ*g/mL). RA-NP were quickly internalized by unchallenged or LPS-treated microglial cells, and their fluorescent signal only began to fade after 24 hours. Previous studies performed by us indicate that this formulation escapes very efficiently the endolysosome compartment [17]. The polymeric components of the nanoparticle after RA release are likely degraded intracellularly in the phagolysosome, an acidified structure containing complex enzymatic machinery that can lead to nanoparticle degradation [34].

Then, we evaluated if RA-NP could shift LPS-induced M1 phenotype (high expression of proinflammatory mediators) to M2 (high levels of anti-inflammatory and neuroprotective factors) [6]. In accordance with previous works reported by us and others, LPS-stimulated NO production [32, 35] and RA-NP were able to prevent NO production and inhibit iNOS expression, thus repressing M1 phenotype. Although free RA (0.4 *μ*M) was also able to reduce NO levels (albeit less robustly than RA-NP), the same treatment was not capable of reverting iNOS expression under an inflammatory challenge. A possible explanation is supported by the constant RA release granted by the formulation [17] (maintaining NO and iNOS levels consistently low), while free RA treatment implies a single pulse (enabling iNOS levels to rise again after RA exhaustion). The slow release of RA achieved by the formulation potentiates the effect of this molecule, as well as protecting it from cytochrome degradation by LPS-activated microglia [36]. These results are in accordance with others that have also shown that RA inhibits iNOS mRNA expression in LPS-activated microglia. This effect was correlated with the high expression of TGF-*β* and the inhibition of nuclear translocation of NF-*κ*B, a transcription factor involved in the inflammatory response (and an inducer of iNOS expression) [14, 32]. Furthermore, RA-NP treatment after LPS challenge led to increased expression of anti-inflammatory mediators (namely, IL-4 and Arg-1), thus promoting an M2 phenotype. M1 microglia will more likely produce cytotoxic NO via iNOS while cells that present an M2 phenotype will produce more ornithine from the same substrate (L-arginine) via arginase, promoting the repair of damaged tissue [37–41]. In sum, the levels of iNOS and Arg-1 affect the inflammatory response in an opposite fashion. Moreover, we showed that RA-NP increased IL-4 expression, under inflammatory conditions. Both RA isoforms (all-*trans* and 9-*cis*) have been shown to promote IL-4 synthesis, while decreasing the production of proinflammatory mediators (IFN-*γ* and IL-12p70, a stimulator of IFN-*γ* and TNF-*α*) by activated human T cells [42]. A recent work has also demonstrated that topical application of 0.1% tretinoin cream (approximately 3 *μ*mol/g RA) significantly enhanced wound healing and that 100 nM RA, in combination with IL-4, activated Arg-1 expression in a macrophage cell line stimulated with 100 ng/mL of LPS [43]. A similar approach that also required the combination of IL-4 plus RA treatment (1 *μ*M) was shown to inhibit the production of proinflammatory cytokines [44]. Ultimately, our RA-NP were more effective *in vitro* than the free equivalent concentration (0.4 *μ*M free RA is the amount found in 10 *μ*g/mL RA-NP). A practical mode of application for RA-NP would be through the intravenous route. However, there is a major obstacle for the delivery of these nanoparticles, namely, the blood-brain barrier (BBB). In that sense, a functionalized formulation (targeting endothelial cells, which can internalize RA-NP) could possibly become a more efficient approach [20, 45]. A BBB-targeted therapy would restrain vascular activation and prevent glia induced-barrier loss [46].

Microglial cells also exhibit distinct morphological changes in a context of neurodegeneration and brain injury. Healthy surveilling microglia leave their highly ramified appearance and retract their processes, which become consequently thicker and fewer, leading to an increasing cell body volume. This physical change is accompanied by the expression of receptors and enzymes, as well as the induction and inhibition of pro- or anti-inflammatory molecules, respectively [3, 47, 48]. With this in mind, we characterized microglial morphology and consequently their activation after LPS challenge and/or RA-NP treatment in organotypic cultures, an ex vivo model that presents complex cellular interactions. Accordingly, we detected a change from activated/amoeboid, with enlarged cell bodies and a low number and length of their processes (LPS-activated state), to surveilling/ramified, with a higher number and length of microglial processes after RA-NP treatment. Others have reported that microglia stimulated with anti-inflammatory molecules, namely IL-4, IL-10 and TGF-*β* exhibit a robust M2 phenotype, which was confirmed by their surveilling/ramified morphology [49]. Here, we report for the first time that RA-NP treatment significantly changed both the activation phenotype *in vitro* and morphology ex vivo of microglial cells, which may play a crucial role on tissue survival and on the development of synaptic contacts. In fact, Vinet and colleagues reported that the adoption of a ramified morphology by microglia in mouse organotypic hippocampal slice cultures rescued neurons from excitotoxic insult [4]. In addition to repress microglia activation, RA-NP were also more efficient than the equivalent free RA concentration showing that this polymeric formulation does not elicit any adverse immune responses. Additionally, cytotoxic agents secreted by microglia are undoubtedly detrimental to neurons by altering their synaptic function [29, 50]. Accordingly, we evaluated if the formulation could reverse LPS-induced neuronal damage. In addition to boosting overall tissue viability, we showed that RA-NP treatment protected neurons from LPS-induced injury by normalizing enolase and PSD-95 levels. Others have shown a correlation between neuroinflammation markers, particularly an increase in IL-1*β*, TNF-*α*, and CD11b levels, and the loss of synaptic proteins [30]. Hence, the clinical reach associated to controlling microglial/macrophage phenotypes is immense. Nevertheless, one should consider that while M2 cells are typically regarded as beneficial, in some particular cases such as cancer therapy, M1 antitumor macrophages are preferable [51, 52].

Since neuroprotection and inflammation are clearly interconnected processes and influence the outcome of one another, RA-NP can be envisioned as a comprehensive approach to the treatment of inflamed brain tissue.

## 5. Conclusions

Herein, we show for the first time that RA-NP inhibit an M1 microglial phenotype while inducing the M2 stage, which ultimately can protect tissue, in particular neurons, from LPS injury and restore the levels of a synaptic function marker. In addition, the formulation was more efficient than the free agent. Thus, RA-NP could open new perspectives for the treatment of several brain inflammatory diseases.
